# Clinical and Molecular Characterization of *PROM1*-Related Retinal Degeneration

**DOI:** 10.1001/jamanetworkopen.2019.5752

**Published:** 2019-06-14

**Authors:** Jasmina Cehajic-Kapetanovic, Johannes Birtel, Michelle E. McClements, Morag E. Shanks, Penny Clouston, Susan M. Downes, Peter Charbel Issa, Robert E. MacLaren

**Affiliations:** 1 Nuffield Laboratory of Ophthalmology, Department of Clinical Neurosciences, Oxford University, Oxford, United Kingdom; 2 Oxford Eye Hospital, Oxford University Hospitals NHS Foundation Trust, Oxford, United Kingdom; 3 Department of Ophthalmology, University of Bonn, Bonn, Germany; 4 Oxford Medical Genetics Laboratories, Oxford University Hospitals NHS Foundation Trust, Oxford, United Kingdom

## Abstract

**Importance:**

The *PROM1* gene*,* commonly associated with cone-rod dystrophies, may have dominant or recessive phenotypes that influence disease onset and severity.

**Objective:**

To characterize the clinical phenotype and molecular genetic variations in patients with *PROM1* variants.

**Design, Setting, and Participants:**

This case-series study was conducted at 2 specialist retinal genetics clinics and examined 19 consecutively enrolled patients with *PROM1-*related retinal degeneration. Data were collected and analyzed from May 2018 to December 2018.

**Main Outcomes and Measures:**

Results of ophthalmic examination, retinal imaging, and molecular genetic analysis by next-generation sequencing.

**Results:**

Of 19 patients, 13 (68%) were women, and age ranged from 11 to 70 years. All patients presented with central visual loss, with or without photophobia. Individuals with recessive variants commonly had severe loss of visual acuity by their 20s, whereas the dominant variant was associated with a milder phenotype, with most patients retaining good vision into late adulthood. The recessive cases were associated with a panretinal dystrophy of cone-rod phenotype with early macular involvement, whereas the dominant variants were associated with a cone-rod phenotype that was restricted to the macula with predominantly cone dysfunction. Next-generation sequencing identified 3 novel and 9 previously reported variants in *PROM1*. Recessive mutations included 6 truncating variants (3 nonsense and 3 frameshift), 4 splice site variants, and 1 missense variant. All 6 dominant variants were associated with a c.1117C>T missense variant. The variants were distributed throughout the *PROM1* genomic sequence with no specific clustering on protein domains.

**Conclusions and Relevance:**

In this case-series study, *PROM1* recessive variants were associated with early-onset, severe panretinal degeneration. The similar phenotypes observed in patients with homozygous missense variants and splice site variants compared with similarly aged patients with truncating variants suggests that all recessive variants have a null (or loss of function close to null) outcome on *PROM1* function. In contrast, the dominant missense cases were associated with a milder, cone-driven phenotype, suggesting that the dominant disease is preferentially associated with cones. This has implications for the development of treatments for this severely blinding disease, and adeno-associated viral vector–based gene therapy and optogenetics could become successful treatment options.

## Key Points

**Question:**

What are the clinical and molecular characteristics of *PROM1-*related retinal degeneration?

**Findings:**

In this case series of 19 patients with *PROM1*-related retinal degeneration, recessive variants were associated with early-onset, severe panretinal degeneration, whereas the dominant disease was associated with the c.1117C>T variant and a late-onset, milder phenotype that predominantly involves the macula. In addition, the dominant variant was preferentially associated with cone photoreceptors.

**Meaning:**

A better understanding of the clinical and molecular characteristics of *PROM1-*related retinal degeneration may aid development of future treatments, including gene therapy and optogenetics.

## Teaser

This case series characterizes the clinical phenotype and molecular genetic variations in patients with retinal degeneration and *PROM1 *sequence variations.

## Introduction

Inherited retinal degenerations are a heterogeneous group of disorders that lead to the progressive degeneration of photoreceptors and loss of vision. On the more severe end of the spectrum lie cone-rod dystrophies, in which there is preferential involvement of the central retina with early manifestations of cone-driven symptoms, including photophobia, dyschromatopsia, reduced visual acuity, and central scotomas. As the disease progresses, there is variable involvement of rods, affecting peripheral and night vision.

Prominin 1 (*PROM1*; OMIM 604365) is commonly associated with cone-rod dystrophies.^1^ However, throughout the literature, variations in *PROM1* have been implicated in extremely varied and overlapping phenotypes that have been described as retinitis pigmentosa^2,3,4,5,6,7,8^; retinitis pigmentosa with macular involvement^9^; retinal dystrophy^8^; cone-rod dystrophy^1,8,10,11,12,13,14,15,16,17^; bull’s-eye maculopathy^18^; bull’s-eye maculopathy associated with rod, rod-cone, and macular dystrophy^19^; Stargardt-like disease^20,21,22,23^; macular dystrophy^24^; and maculopathy with rod-cone dystrophy.^25^ The age at onset, presenting symptoms, and severity of disease vary with sequence variations. Moreover, variants can be inherited in recessive and dominant fashions.^15^

Despite many genetic reports that focus on identifying *PROM1* variants, the genotype-phenotype correlation remains poorly understood. In addition, the molecular roles of these sequence variations and their effects on protein function are largely unknown. Moreover, it remains unclear if *PROM1* has a more important role in cone or rod photoreceptors and how variants in the same gene can have a recessive and/or dominant effect. The aim of this study is to analyze the sequence variations in the *PROM1* gene in a series of patients to understand better their potential for pathophysiological consequences on retinal degeneration. This has important implications in the development of potential gene-based therapies and optogenetics for this complex genetic condition, especially as the successful treatment of macular photoreceptors could be sufficient to preserve good visual acuity in affected individuals.

## Methods

### Clinical Assessment and Imaging

The study design adhered to the tenets of the Declaration of Helsinki.^26^ This study follows the reporting guideline for case series. Institutional review boards at Oxford University Hospitals and the University of Bonn approved the studies, and patients provided written informed consent.

Patients were identified from genetic databases between July 1, 2014, and May 1, 2018, at 2 clinical genetic centers in Oxford, United Kingdom, and Bonn, Germany. The data analysis was conducted from May 1, 2018, to December 1, 2018. Medical records of patients with *PROM1 *variants were reviewed for information on family, general history, and ophthalmic history. Details of clinical assessments, including visual acuity and dilated fundal examinations, were collected. Retinal imaging studies, including color photography, fundus autofluorescence (55° and 30°), and optical coherence tomography, were taken with the Heidelberg Spectralis system (Heidelberg Engineering). In addition, widefield fundus imaging was performed with the Optos 200Tx confocal scanning laser ophthalmoscopy camera (Optos).

### Genetic Analysis

Sequence variations in *PROM1* were identified by targeted next-generation sequencing techniques. For patients at the Oxford site, enrichment for *PROM1* was achieved as part of a customized HaloPlex enrichment system kit (Agilent Technologies) designed to capture the coding exons and 10 base pairs of the flanking introns of 117 retinal genes. HaloPlex reactions were prepared per manufacturer’s instructions. Libraries were pooled into batches of 14 and sequenced on an Illumina MiSeq instrument (Illumina) using a MiSeq version 3 kit per manufacturer’s instructions. Reads were aligned using Burrows-Wheeler Aligner 2,^27^ and variants were called using Platypus.^28^ All variants identified by next-generation sequencing were confirmed by Sanger sequencing. For patients at the Bonn site, the next-generation sequencing methodology was performed as described previously.^1^

Sequence variations were assessed for pathogenic association with protein function based on in silico analysis using Alamut Visual (Alamut Interactive Biosoftware). Nonsense variants and frameshift variants were considered pathogenic unless at the 3′ end of the gene. The likely pathogenicity of missense variants was crosschecked against the PolyPhen-2, SIFT, and Grantham matrix (for those variants that led to a stop codon) algorithm scores. The allelic frequency of variants was evaluated in gnomAD, which includes the Exome Aggregation Consortium data set. Geneious bioinformatics software version 11.1.5 (Geneious) was used for the *PROM1* nucleotide sequence analysis and mapping of specific variants to protein domains. For patients with autosomal recessive inheritance patterns and compound heterozygous variants (6 of 13 patients [46%]), no further genotyping analysis was carried out in individuals’ parents to exclude the rare possibility that the 2 variants were present on the same chromosome.

## Results

Overall, 19 patients (13 [68%] women) aged 11 to 70 years were included in this study. Inheritance was autosomal recessive in 13 patients (68%) and autosomal dominant in 6 patients (32%). Ethnic origins included 11 British patients (58%), 5 German patients (26%), 2 Turkish patients (11%), and 1 Ukrainian patient (5%). Details of participant demographic characteristics, history, clinical findings, and genotype analysis are summarized in the Table. Most participants (17 [89%]) were unrelated, except AD4 and AD5, who are related.

**Table.** Demographic Characteristics and Phenotype-Genotype Correlation in Patients With *PROM1* Variants

Patient^**a**^	Sex	Age, y	Age at Onset, y	Presenting Symptoms (Duration of Symptoms, y)	Visual Acuity, logMAR	Severity of Phenotype	Other Ophthalmic Features	Genotype	*PROM1* Variant(s)	Predicted Amino Acid Sequence Change(s)	Variant Effect(s)
AR1	F	60s	20	Photophobia, central visual loss (44)	HM OD; LP OS	Severe panretinal dystrophy	Cataract in RE; IOL in LE	Compound heterozygous	c.1557C>A	p.Tyr519*	Nonsense
									c.1177_1178delAT	p.Ile393Argfs*21	Frameshift
AR2	M	50s	12	Photophobia, central visual loss (40)	HM OU	Severe panretinal dystrophy	Oscillopsia; cataract in RE; IOL in LE	Compound heterozygous	c.1354_1355insT	p.Tyr452Leufs*13	Frameshift
									c.22del^b^	p.Leu8fs*	Frameshift
AR3	F	50s	14	Central visual loss (40)	LP OU	Severe panretinal dystrophy	Nystagmus; IOL and retinal implant in RE; cataract in LE	Compound heterozygous	c.1354_1355insT	p.Tyr452Leufs*13	Frameshift
									c.436C>T	p.Arg146*	Nonsense
AR4	M	50s	8	Photophobia, central visual loss (44)	LP OU	Severe panretinal dystrophy	PSCLO in BE	Compound heterozygous	c.1354_1355insT	p.Tyr452Leufs*13	Frameshift
									c.1557C>A	p.Tyr519*	Nonsense
AR5	F	40s	6	Central visual loss (41)	1.6 OD; 1.4 OS	Severe panretinal dystrophy	Myopia; nystagmus; PSCLO in BE	Homozygous	c.1354_1355insT	p.Tyr452Leufs*13	Frameshift
AR6	M	20s	Childhood	Central visual loss, scotoma (>20)	1.3 OD; 1.3 OS	Severe panretinal dystrophy	Myopia; nystagmus	Homozygous	c.199C>T	p.Gln67*	Nonsense
AR7	F	30s	16	Photophobia, daylight vision problems (22)	HM OU	Severe panretinal dystrophy	Cataract in RE; IOL in LE	Compound heterozygous	c.1301+2T>C^b^	Splice donor site	Aberrant splicing
									c.1767G>A^b^	Splice donor site	Aberrant splicing
AR8	M	50s	28	Central visual loss, slow dark adaptation (30)	LP OU	Severe panretinal dystrophy	None	Homozygous	c.1579-1G>C	Splice acceptor site	Aberrant splicing
AR9	F	20s	18	Central visual loss (7)	1.7 OD; 1.6 OS	Severe panretinal dystrophy	Myopia; nystagmus; PSCLO in BE	Homozygous	c.1142-1G>A	Splice acceptor site	Aberrant splicing
AR10	F	20s	Childhood	Central visual loss, nyctalopia (>20)	LP OU	Severe panretinal dystrophy	Myopia; PSCLO in BE	Homozygous	c.1853T>G	p.Leu618Arg	Missense
AR11	F	<18	Childhood	Central visual loss (>5)	0.2 OD; 0.3 OS	Mild panretinal dystrophy	Strabismus in LE	Homozygous	c.1142-1G>A	Splice acceptor site	Aberrant splicing
AR12	F	30s	Childhood	Central visual loss (>25)	CF OU	Severe panretinal dystrophy	None	Compound heterozygous	c.1354_1355insT	p.Tyr452Leufs*13	Frameshift
									c.1142-1G>A	Splice acceptor site	Aberrant splicing
AR13	M	60s	40	Central visual loss (20)	CF OD; 0.2 OS	Moderate macular dystrophy	None	Homozygous	c.1354_1355insT	p.Tyr452Leufs*13	Frameshift
AD1	F	70s	55	Central visual loss, scotoma (25)	1.0 OD; 0.0 OS	Mild to moderate macular dystrophy	None	Heterozygous	c.1117C>T	p.Arg373Cys	Missense
AD2	M	50s	40	Photophobia, central visual loss (14)	0.2 OD; 0.6 OS	Mild macular dystrophy	None	Heterozygous	c.1117C>T	p.Arg373Cys	Missense
AD3	F	50s	42	Photophobia, central visual loss (10)	0.4 OU	Mild macular dystrophy	None	Heterozygous	c.1117C>T	p.Arg373Cys	Missense
AD4	F	50s	41	Central visual loss (12)	1.0 OU	Moderate macular dystrophy	None	Heterozygous	c.1117C>T	p.Arg373Cys	Missense
AD5	F	30s	29	Central visual loss, scotoma (2)	0.1 OD; 0.0 OS	Mild macular dystrophy	None	Heterozygous	c.1117C>T	p.Arg373Cys	Missense
AD6	F	30s	25	Central visual loss, scotoma (5)	1.00 OD; 0.8 OS	Mild macular dystrophy	None	Heterozygous	c.1117C>T	p.Arg373Cys	Missense

Abbreviations: AD, autosomal dominant; AR, autosomal recessive; BE, both eyes; CF, counting fingers; F, female; HM, hand movements; IOL, intraocular lens implant; LE, left eye; LP, light perception; M, male; PSCLO, posterior subcapsular lens opacity; RE, right eye.

^a^ First 2 letters of ID indicate variant inheritance pattern (recessive or dominant).

^b^ Previously unreported variant.

**Figure 1.**
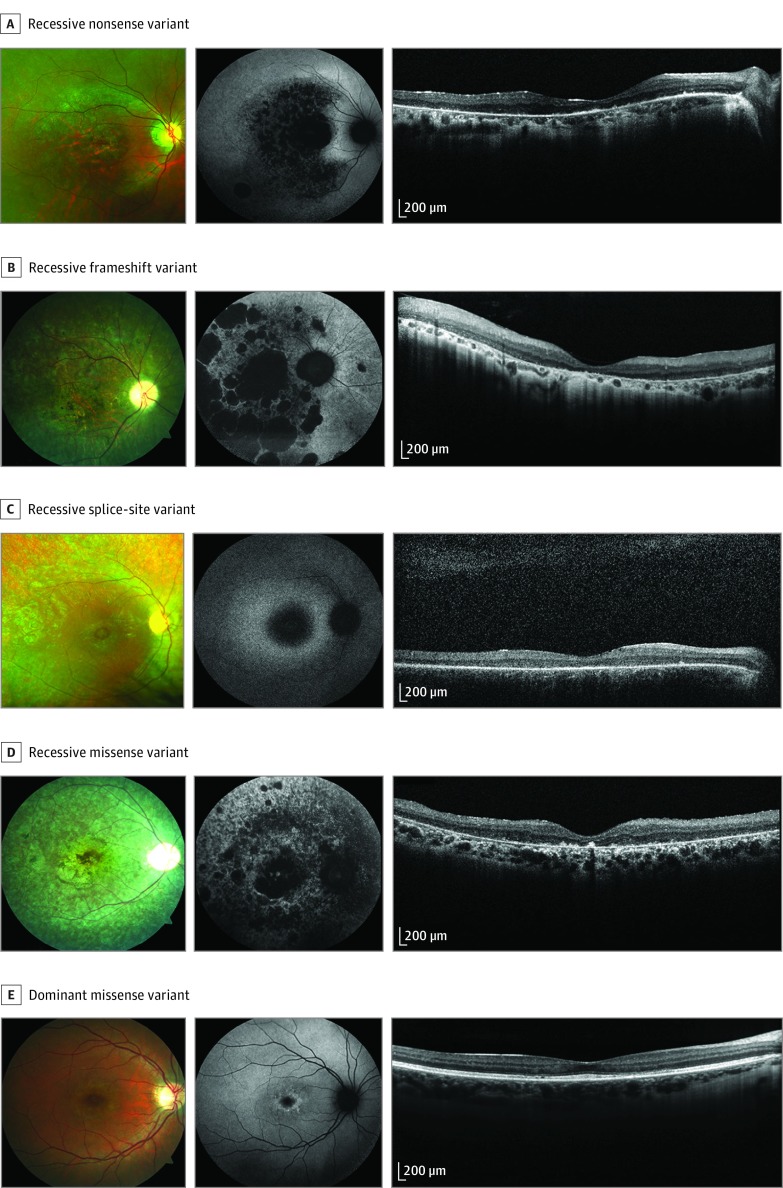
Representative Images of *PROM1-*Related Retinal Degeneration Associated With Recessive and Dominant Genotypes From left to right, images are color photographs, fundus autofluorescence photographs, and optical coherence tomography images. A, Images of patient AR6; visual acuity, 1.3 OD and 1.3 OS; c.199C>T variant. B, Images of patient AR5; visual acuity, 1.6 OD and 1.4 OS; c.1354dup variant. C, Images of patient AR9; visual acuity, 1.7 OD and 1.6 OS; c.1142-1G>A variant. D, Images of patient AR10; visual acuity, light perception in both eyes; c.1853T>G variant. E, Images of patient AD5; visual acuity, 0.1 OD and 0.0 OR; c.1117C>T variant.

The clinical phenotypes of recessive and dominant variant classes are shown in Figure 1. Independent of variant class (ie, nonsense, frameshift, splice site, and missense), recessive genotypes showed severe panretinal dystrophy with macular involvement and peripheral pigment spicules. Fundus autofluorescence showed areas of macular hypoautofluorescence with variable degrees of patchier hypoautofluorescence patterns in the periphery. Optical coherence tomography revealed widespread loss or thinning of the outer retina in all recessive genotypes. In contrast, at a comparable age, the dominant genotype showed much milder changes, largely restricted to the macula on fundus autofluorescence and optical coherence tomography imaging, and no peripheral bone spicule pigmentations.

**Figure 2.**
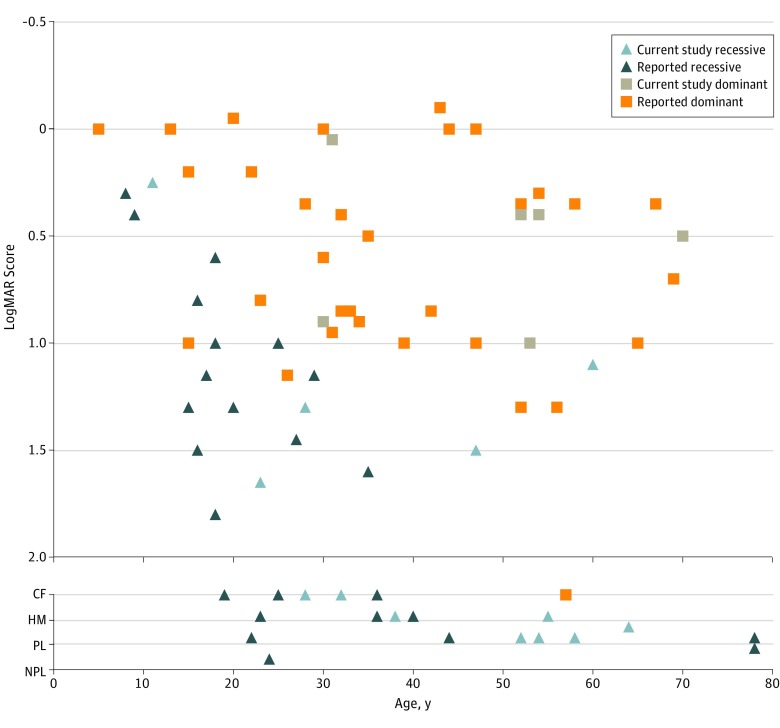
Association of Visual Acuity With Age at Time of Presentation in All *PROM1* Variants Reported to Date The mean logMAR score between the right and left eyes is plotted against age. CF indicates counting fingers; HM, hand movements; NPL, no perception of light; and PL, perception of light.

The age at onset of retinal degeneration ranged from early childhood (recessive genotype) to 40 years (dominant genotype), with all patients presenting with central visual loss, indicating early cone involvement. Photophobia was present in 6 patients (32%) with recessive and dominant genotypes. The association of visual acuity with patient age is depicted in Figure 2 and eTable 1 in the Supplement, both of which include, to our knowledge, all *PROM1* cases reported with sufficient data to date. There is a decline in visual acuity in recessive variants from an early age, with visual acuity of 1.0 logMAR (Snellen equivalent, 20/200) or less in patients older than 20 years, and most patients having visual acuity of counting fingers or less after the age of 30 years. In contrast, dominant *PROM1* mutations in this series were associated with a later onset and a slower decline in visual function, with most patients having visual acuity of at least 1.0 logMAR at the time of presentation. (Three of 6 patients with the dominant variant were in their 50s.) Previously reported^19,24^ dominant *PROM1*-related dystrophies (2 variants, c.1117C>T [31 patients] and c.2485G>A [1 patient]) show a similar trend.

Other ophthalmic features appeared in patients with recessive variations including myopia (4 patients [21%]), cataract or history of cataract extraction (8 patients [42%]), nystagmus (4 patients [21%]), oscillopsia (1 patient [5%]), and strabismus (1 patient [5%]). There were no consistently associated systemic diseases, although 1 patient had polymyalgia rheumatica and another had psoriasis.

We identified 12 mutations in *PROM1* that may be associated with the disease phenotype, of which 3 were novel (Table) (eTable 2 in the Supplement). All patients with dominant inheritance had the known c.1117C>T missense variant in *PROM1*. In patients with autosomal recessive disease, 6 variants were truncating variants (3 nonsense and 3 frameshift), 4 were splice site variants, and 1 was a missense variant (Table). Overall, 7 of 13 patients with recessive disease were homozygous for the identified *PROM1* variant.

Detailed in silico analysis confirmed a pathogenic association with protein function, with all sequence variations having a highly likely pathogenic effect, including the supporting evidence for missense variants from the PolyPhen2, SIFT, and Grantham matrix algorithm scores (eTable 2 in the Supplement). The novel variants were not previously reported in the literature, and they were crosschecked against normal variants based on the gnomAD dataset (eTable 2 in the Supplement).

**Figure 3.**
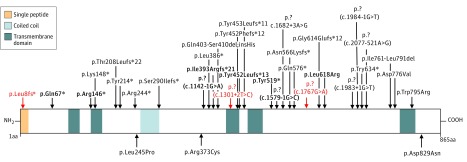
Schematic Representation of *PROM1* and Associated Variants The *PROM1* protein is shown as a white bar with the respective protein domains depicted in different colors. Recessive variants are shown above, and dominant variants are shown below. Variants from the current study appear in bold, and novel variants appear in red. For splice site and frameshift variants, the arrow indicates the location of the first affected amino acid. Variants without a reliable prediction on the protein (eg, splice site) are marked as p.? with their c. nomenclatures shown underneath.

A schematic diagram of all *PROM1* mutations reported to date depicts their distribution throughout the protein, with no specific clustering relative to protein domains (Figure 3) (eTable 1 in the Supplement). Most *PROM1* variants (34 of 41 reported with known inheritance) were found to be recessive (31 of 34: 11 nonsense variants, 9 frameshift variants, 8 splice site variants, and 3 missense variants), whereas only 3 of 34 were dominant (all missense variants). The dominant cases in the current study had the same variant (p.Arg373Cys). The inheritance pattern of reported variant p.Leu3fs*^15^ remains unclear (excluded from Figure 3) (shaded in green in eTable 1 in the Supplement). The inheritance pattern of 6 variants is not available in the reporting studies^8,23^ (excluded from Figure 3) (shaded in yellow in eTable 1 in the Supplement).

## Discussion

In this case-series study, we analyzed the sequence variations in the *PROM1* gene, and we identified 3 novel sequence variations. The types of variant as well as the mode of inheritance were associated with disease phenotype, age at onset of symptoms, and severity of retinal degeneration.

The *PROM1* gene encodes a pentaspan transmembrane domain glycoprotein,^29^ which is expressed ubiquitously in plasma membrane protrusions. It is best known as a surface marker of endothelial progenitor, hematopoietic stem cells (AC133 and CD133) and cancer cells in the central nervous system. In retinal photoreceptors, it has a critical structural role. The protein specifically localizes to membrane protrusions at the base of rod and cone outer segments, where it plays a key role in disc morphogenesis^30^ and subsequent photopigment sorting.^31^ More recently, *PROM1* has been found to be associated with the regulation of photoreceptor autophagy in retinal pigment epithelium cells.^32^

Given the ubiquitous expression of *PROM1*, a remarkable feature of *PROM1*-associated retinal dystrophy in the current series was the absence of an extraocular phenotype. We found no syndromic cases and 2 systemic conditions that were unlikely to be associated with *PROM1* expression. This is in keeping with the current literature, with only 1 study reporting a family with the p.Arg373Cys variant having some subclinical systemic features.^33^ Despite the rarity of an observed extraocular *PROM1* phenotype, we nonetheless need be aware of possible systemic manifestations of the *PROM1* variant when faced with such patients in a genetic clinic. In our study, we noted that, among other ocular features, one-third of patients had myopia. Although it is plausible that *PROM1* is implicated in the pathogenicity of myopia, a more likely explanation is that the myopia is induced by sensory deprivation owing to blurring of vision in patients with *PROM1 *sequence variations.

Although previous genetic reports have described numerous *PROM1*-associated retinal dystrophy phenotypes,^1,2,3,4,5,6,7,8,9,10,11,12,13,14,15,16,17,18,19,20,21,22,23,24,25^ in the current study, we found that the morphological phenotype was associated with cone-rod dystrophy in all cases. The main distinction in phenotypes lies between recessive and dominant forms of the disease. The recessive disease was associated with early-onset, severe panretinal degeneration with early central loss of vision, whereas the dominant disease was associated with late-onset dystrophy predominantly involving the macula. In contrast to some previously reported cases,^2,3^ we did not find any patients who presented with night vision problems that would suggest early rod-driven functional deficiencies. It is possible, owing to the relatively fast progression of *PROM1*-associated recessive cone-rod dystrophy, that cone and rod dysfunction occur in close succession or simultaneously, making it difficult for patients to distinguish which symptoms (ie, central blurring of vision or nyctalopia) came first. In addition, as the disease progresses, pigmentation indistinguishable from rod-cone dystrophy appears in the retinal periphery.

Our analysis of *PROM1* variations showed that the entire protein is associated with sequence variations, with no major clustering in a domain-dependent manner, suggesting that the entire protein is important to its function. The severe homozygous recessive phenotype is likely associated with null variants that abolish the function of alleles, leading to the absence of *PROM1*. Most frameshift and nonsense variants result in a premature stop codon, leading to a truncated modified RNA that is quickly degraded by nonsense-mediated decay before undergoing translation.^2^ In addition, the missense and splice site variants involved in the recessive disease are likely associated with a null (or loss of function close to null) effect, as evident from the similar phenotypes in our patients with homozygous missense and splice site variants compared with similarly aged patients with truncating mutations. These loss-of-function variants are associated with disorganized optic disc membranes and photoreceptor degeneration, as shown in *PROM1* knockout mice.^31^

**Figure 4.**
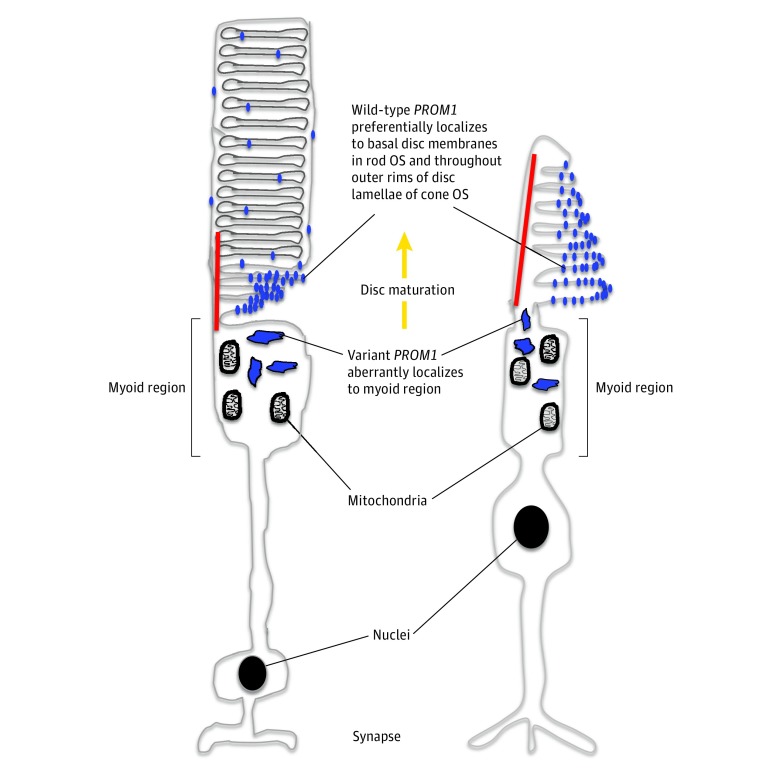
Schematic Diagram of Rod and Cone Photoreceptors, Depicting Localization of Wild-Type and Variant *PROM1* OS indicates outer segment.

In contrast, a milder, dominant phenotype, as observed in our study as well as in most other studies, is associated with a dominant negative effect of a missense variant. This appears to result in a stable variant protein that is associated with the mislocalization of the variant protein. It also appears to interfere with the function of the wild-type protein as well as *CDHR1 *and actin,^30^ exerting a dominant negative effect. As cysteine residues are evolutionarily conserved in *PROM1*, it is possible that the addition of another cysteine residue in p.Arg373Cys disrupts disulfide bridges, impairing homophilic protein interactions. The cone-associated phenotype observed in the dominant disease suggests that the dominant variant is preferentially associated with cones. A 2008 study^30^ that implicates *PROM1* in disc morphogenesis used a transgenic mouse model carrying the Arg373Cys variant expressed in rods only under the control of rhodopsin promoter, thereby making it difficult to study the direct effect of this variant in cones. However, in a *PROM1* knockout mouse model,^31^ the null protein impairs disc morphogenesis as well as causes ectopic accumulation of visual pigment in rods and cones. Since cone opsins are more prone to mislocalization than rod opsins,^34^ this could explain why cones become affected first and with greater severity in *PROM1* degeneration. In addition, there is some evidence of a differential distribution of wild-type *PROM1* protein between rods and cones.^29,35^ In contrast to rods, the cones’ outer segment lamellar membrane is suggested to have a wide distribution of *PROM1* (Figure 4). The open lamellar structure of cones’ outer segments makes cone proteins more exposed to extracellular space and to interference from other proteins, predisposing cones to premature degeneration.

Clarifying the mechanisms underlying *PROM1* degeneration has important implications in the development of potential treatments. Subretinal delivery of adeno-associated viral vector–carrying *PROM1* (2.5 kilobase pairs) to photoreceptors at an early stage of recessive dystrophy could replace the null protein and potentially rescue the phenotype. However, the dominant variant would need to be silenced first, eg, RNA silencing via a mirtron,^36^ followed by a gene replacement therapy of the wild-type protein, ie, block-and-replace therapy. In more advanced instances of disease, where irreversible photoreceptor damage has occurred, restoring sight by optogenetic treatment^37^ may be an alternative therapy. Moreover, the ubiquitous expression of *PROM1* will facilitate the development of in vitro models of *PROM1* gene therapy and related functional assays.

### Study Limitations

This noninterventional case series of 19 patients with *PROM1* variants is limited by the retrospective, uncontrolled nature of the study. In our series and in other reports (with the exception of 2 cases), the dominant disease was caused by a single c.1117C>T variant. Nonetheless, our study, which, to our knowledge, is the largest reported *PROM1* case series to date, adds substantially to the knowledge and understanding of *PROM1*-related retinal disease and possible future therapies. Further longitudinal studies will shed more light on the natural progression of the disease and the timing of therapeutic interventions.

## Conclusions

To our knowledge, this is the largest reported case series on *PROM1*-related retinal degeneration to date. This study shows that *PROM1* recessive variants were associated with early-onset, severe retinal degeneration, whereas the c.1117C>T variant, which was associated with autosomal dominant inheritance, showed a milder, cone-driven phenotype. This clinical and molecular characterization has deepened understanding of the disease and will aid in the design of future randomized clinical trials and therapeutic approaches.

## Notes

Supplement. **eTable 1.** *PROM1* Variants Reported to Date Including Their Respective References **eTable 2.** Detailed Description of Sequence Variations in *PROM1* in the Series With Predicted Effect on Protein Function
