# Supplementary material for: Clinical and Molecular Characterization of PROM1-Related Retinal Degeneration
Source: JAMA Netw Open. 2019 Jun 14;2(6):e195752. doi: 10.1001/jamanetworkopen.2019.5752 (PMC6575153; doi:10.1001/jamanetworkopen.2019.5752)
Supplement: Supplement. — eTable 1. PROM1 Variants Reported to Date Including Their Respective References eTable 2. Detailed Description of Sequence Variations in PROM1 in the Series With Predicted Effect on Protein Function [file jamanetwopen-2-e195752-s001.pdf]

Supplementary Online Content

Cehajic-Kapetanovic J, Birtel J, McClements ME, et al. Clinical and molecular characterization of *PROM1*-related retinal degeneration. *JAMA Netw Open*. 2019;2(6):e195752. doi:10.1001/jamanetworkopen.2019.5752

eTable 1. *PROM1* Variants Reported to Date Including Their Respective References
eTable 2. Detailed Description of Sequence Variations in *PROM1* in the Series With Predicted Effect on Protein Function

This supplementary material has been provided by the authors to give readers additional information about their work.

© 2019 Cehajic-Kapetanovic J et al. *JAMA Network Open.*

eTable 1. *PROM1* Variants Reported to Date Including Their Respective References

PROM1 variant	Amino acid change	Reference	Visual Acuity Snellen RE, LE (LogMAR) (Age, years)
c.7dup	p.Leu3fs*	Boulanger-Scemama et al. 2015	Not reported
c.22del	p.Leu8fs*	Current study	HM, HM, (55) #ar2
c.199C>T	p.Gln67*	Current study<br>Birtel et al. 2018	6/120 (1.3), 6/120 (1.3), (28) #ar6<br>Same patient as #ar6
c.436C>T	p.Arg146*	Current study<br>Birtel et al. 2018	PL, PL, (54) #ar3<br>Not reported
c.442A>T	p.Lys148*	Jinda et al. 2014	HM, HM, (23)
c.622delA	p.Thr208Leufs22*	Zhao et al. 2015	PL, NPL (78)
c.630_c630+8del9ins18	p.?	Strauss et al. 2018	Not reported
c.642T>A	p.Tyr214*	Eisenberger et al. 2013	Not reported
c.730C>T	p.Arg244*	Zhang et al. 2014<br>Carss et al. 2017	6/60 (1.0), 6/60 (1.0) (25)<br>Not reported
c.734T>C	p.Leu245Pro	Imani et al. 2018	Not reported (63)<br>(tunnel vision and reduced central vision but no values)
c.869delG	p.Ser290Ilefs*	Permanyer et al. 2010	PL, 6/60 (1.0) (35)<br>6/240 (1.6), 6/120 (1.3) (27)<br>6/30 (0.7), 6/19 (0.5) (18)
c.963G>T	pLeu321Phe	Strauss et al. 2018	Not reported
c.1002+5G>A	p.?	Carss et al. 2017	Not reported
c.1117C>T	p.Arg373Cys	Current study<br>Birtel et al. 2018<br>Yang et al. 2008<br>Kniazeva et al. 1999<br>Michaelides et al. 2010	6/60 (1.0), 6/6 (0.0), (70) #ad1<br>6/9 (0.2), 6/24 (0.6), (54) #ad2<br>6/15 (0.4), 6/15 (0.4), (52) #ad3<br>6/60 (1.0), LE 6/60 (1.0), (53) #ad4<br>6/7.5 (0.1), 6/6 (0.0), (31) #ad5<br>6/60 (1.0), 6/38 (0.8), (30) #ad6

© 2019 Cehajic-Kapetanovic J et al. *JAMA Network Open.*

			Same patients as #ad4 and #ad5<br><br><br>6/24 (0.6), 6/24 (0.6), age not reported<br><br>20/400 (1.3), 20/50 (0.4), (33)<br><br>6/60 (1.0), 6/60 (1.0), (47)<br>6/120 (1.3), 6/120 (1.3), (52)<br>CF, CF, (57)<br>6/60 (1.0), 6/60 (1.0), (15)<br>6/6 (0.0), 6/7.5 (0.1), (30)<br>6/9 (0.2), 6/9 (0.2), (22)<br>6/24 (0.6), 6/24 (0.6) (30)<br>6/120 (1.3, congenital cataract), 6/45 (0.9),(31)<br>6/18 (0.5), 6/60 (1.0), (35)<br>6/48 (0.9), 6/48 (0.9), (34)<br>6/120 (1.3), 6/15 (0.4), (32)<br>6/6 (0.0), 6/6 (0.0), (13)<br>6/6 (0.0), 6/6 (0.0), (5)<br>6/24 (0.6), 6/36 (0.8), (69)<br>6/18 (0.5), 6/9 (0.2), (67)<br>6/6 (0.0), 6/6 (0.0), (47)<br>6/5 (-0.1), 6/5 (-0.1), (43)<br>6/36 (0.8), 6/36 (0.8), (23)<br>6/9 (0.2), 6/9 (0.2), (15)<br>6/6 (0.0), 6/5 (-0.1), (20)<br>6/12 (0.3), 6/15 (0.4), (58)<br>6/120 (1.3), 6/120 (1.3), (56)<br>6/15 (0.4), 6/12 (0.3), (52)

© 2019 Cehajic-Kapetanovic J et al. *JAMA Network Open.*

			6/60 (1.0), 6/30 (0.7), (42)<br>6/60 (1.0), 6/60 (1.0), (39)<br>6/120 (1.3), 6/60 (1.0), (26)<br>6/60 (1.0), 6/60 (1.0), (65)<br>6/18 (0.5), 6/12 (0.3), (32)<br>6/6 (0.0), 6/6 (0.0), (44)<br>6/12 (0.3), 6/6 (0.3), (54)
c.1142–1G>A	p.?	Current study<br>Littink et al. 2010	6/300(1.7), 6/240 (1.6), (25) #ar9<br>6/9 (0.2), 6/12 (0.3), (11) #ar11<br>CF, CF, (32) #ar12<br>6/38 (0.8; only better seeing eye reported), (16)<br>6/60 (1.0; only better seeing eye reported), (18)
c.1157T>A	p.Leu386*	Beryozkin et al. 2014	PL, PL, (22)<br>CF, CF, (36)
c.1177_1178delAT	p.Ile393Argfs*21	Current study<br>Carss et al. 2017	HM, PL, (64) #ar1<br>Not reported
c.1209–1229del	p.Gln403-Ser410del;insHis	Eisenberger et al. 2013	Not reported
c.1301+2T>C	p.?	Current study	HM, HM, (38) #ar7
c.1349insT	p.Tyr452Phefs*12	Pras et al. 2009	CF, CF, (19)<br>CF, CF, (25)<br>6/120 (1.3), 6/60 (1.0), (29)
c.1354_1355insT	p.Tyr452Leufs*13	Current study<br>Birtel et al. 2018<br>Crass et al. 2017	HM, HM (55) #ar2<br>PL, PL, (54) #ar3<br>PL, PL, (52) #ar4<br>6/240 (1.6), 6/150 (1.4), (47) #ar5<br>CF, CF (32) #ar12

© 2019 Cehajic-Kapetanovic J et al. *JAMA Network Open.*

			CF, 6/9 (0.2), (60) #ar13<br>Same patient as #ar5<br>Not reported
c.1355_1356insT	p.Tyr453Leufs*11	Zhao et al. 2015	PL, NPL, (78)
c.1557C>A	p.Tyr519*	Current study<br><br>Song et al. 2011<br>Crass et al. 2017	HM, PL, (64) #ar1<br>PL, PL (52) #ar4<br>Not reported<br>Not reported
c.1579–1G>C	p.?	Current study<br>Boulanger-Scemama et al. 2015<br>Crass et al 2017	PL, PL, (58) #ar8<br>Not reported<br><br>Not reported
c.1682+3A>G	p.?	Liang et al. 2019	CF, PL (41)<br>CF, CF (32)
c.1697dupA	p.Asn566Lysfs*	Khan et al. 2015	2/60 (1.4), 4/60 (1.2), (20)
c.1697delA	p.Asn566Ilefs*	Strauss et al. 2018	Not reported
c.1710C>A	p.Tyr570*	Crass et al. 2017	Not reported
c.1726C>T	p.Gln576*	Zhang et al. 2007<br>Carss et al. 2017	PL, PL, (44)<br>HM, PL, (40)<br>HM, PL, (36)<br>6/360 (1.75), 6/360 (1.75), (18)<br>6/60 (1.0), HM, (16)<br>6/120 (1.3), 6/120 (1.3), (15)<br>Not reported
c.1767G>A	p.?	Current study	HM, HM, (38) #ar7
c.1767+4A>G	p.?	Strauss et al. 2018	Not reported
c.1841delG	p.Gly614Glufs*12	Maw et al. 2000	Not available;<br>Full paper not on PubMed<br>(Third decade, profound visual impairment, but no detail)

© 2019 Cehajic-Kapetanovic J et al. *JAMA Network Open.*

c.1853T>G	p.Leu618Arg	Current study<br>Birtel et al. 2018	PL, PL, (28) #ar10<br>Same patient as #ar10
c.1983+1G>T	p.?	Zhang et al. 2014	6/60 (1.0), 6/60 (1.0) (25)
c.1984–1G>T	p.?	Boulanger-Scemama et al. 2015	Not reported
c.1902C>G	p.Tyr634*	Liang et al. 2019	CF, PL (41)<br>CF, CF (32)
c.2077–521A>G	p.?	Mayer et al. 2016	Not reported
c.2281–26_-17del	p.Ile761-Leu791del	Eidinger et al. 2015	6/60 (1.0), 3/60 (1.3), (17)<br>6/12 (0.3), 6/12 (0.3), (8)<br>6/12 (0.3), 6/20 (0.5), (9)
c.2327A>T	p.Asp776Val	Salles et al. 2017	NPL, NPL, (24)
c.2383T>C	p.Trp795Arg	Boulanger-Scemama et al. 2015	Not reported
c.2485G>A	p.Asp829Asn	Salles et al. 2017	6/12 (0.3), 6/15 (0.4), (28)

Presenting visual acuity (Snellen and LogMar) and patients' age where available is included for each *PROM1* mutation. Dominant mutations are shaded in gray. A mutation of uncertain inheritance is shaded in green. A mutation of unreported inheritance is shaded in yellow. Novel mutations are shaded in blue. # Patient ID as per Table 1. RE – right eye. LE – left eye. CF – counting fingers. PL – perception of light. NPL – no perception of light.

© 2019 Cehajic-Kapetanovic J et al. *JAMA Network Open.*

eTable 2. Detailed Description of Sequence Variations in *PROM1* in the Series With Predicted Effect on Protein Function

**Nucleotide sequence (EXON)**	**Predicted amino acid change**	**Allele frequency (gnomAD browser)**	**SIFT Score; Poly-Phen 2 Score**	**Grantham matrix score**	**Mutation prediction**	**Predicted effect on protein function**	**Pathogenicity**	**ACMG classification**	**Reference**
c.22del (1)	p.Leu8fs*	Not available	-	-	frameshift	Premature termination of translation/null effect	Highly likely	Pathogenic	Novel
c.199C>T (1)	p.Gln67*	Not available	-	6	stop gained	Premature termination of translation/null effect	Highly likely	Pathogenic	Birtel et al 2018
c.436C>T (4)	p.Arg146*	0.0012	-	6	stop gained	Premature termination of translation/null effect	Highly likely	Pathogenic	Birtel et al 2018,<br>Carss et al 2017

© 2019 Cehajic-Kapetanovic J et al. *JAMA Network Open.*

c.1117C>T (10)	p.Arg373Cys	Not available	0.11 (predicted to be tolerated); 0.936 (possibly damaging)	180	missense	Protein misslocalisation in myoid region of photoreceptors	Highly likely	Pathogenic	Birtel et al 2018, Carss et al 2017, Michaelides et al 2010, Yang et al 2008, Kniazeva et al. 1999
c.1142-1G>A (11)	Splice site	0.0032	-	-	splice acceptor lost	Abberant splicing	Highly likely	Pathogenic	Littink et al 2010
c.1177_1178delAT (11)	p.Ile393Argfs*21	0.0012	-	-	frameshift	Premature termination of translation/null effect	Highly likely	Pathogenic	Carss et al 2017
c.1301+2T>C (11)	Splice site	0.0036	-	-	splice donor lost	Abberant splicing	Highly likely	Pathogenic	Novel

© 2019 Cehajic-Kapetanovic J et al. *JAMA Network Open.*

c.1354_1355insT (12)	p.Tyr452Leufs*13	0.021	-	-	frameshift	Premature termination of translation/null effect	Highly likely	Pathogenic	Birtel et al 2018
c.1557C>A (13)	p.Tyr519*	0.0039	-	6	stop gained	Premature termination of translation/null effect	Highly likely	Pathogenic	Song et al 2011
c.1579-1G>C (14)	Splice site	0.003	-	-	splice acceptor lost	Abberant splicing	Highly likely	Pathogenic	Boulanger-Scemama et al 2015
c.1767G>A (15)	Splice site	0.0004	-	-	splice donor lost	Abberant splicing	Highly likely	Pathogenic	Novel

© 2019 Cehajic-Kapetanovic J et al. *JAMA Network Open.*

c.1853T>G (16)	p.Leu618Arg	Not available	0.00 (predicted to affect protein function); 01.000 (probably damaging)	102	missense	Protein misslocalisation in myoid region of photoreceptors	Highly likely	Pathogenic	Birtel et al 2018

Mutations were predicted to have a pathogenic effect on protein function based on *in silico* analysis using Alamut Visual (Alamut Interactive Biosoftware, Rouen, France). Putative pathogenic variants were confirmed by Sanger sequencing. Nonsense and frameshift variants were considered to be pathogenic, unless at the 3' end of the gene. The likely pathogenicity of missense variants was crosschecked against the Polymorphism Phenotyping version 2 (PolyPhen-2), SIFT and Grantham Matrix (for those mutations that led to a stop codon) algorithm scores. The 2015 American College of Medical Genetics and Genomics (ACMG) recommendations on interpretation of variants is also included.[1] The novel mutations (shaded in blue) were not previously reported in the literature and they were crosschecked against normal variants based on gnomAD dataset.

1. Richards S, Aziz N, Bale S, et al; ACMG Laboratory Quality Assurance Committee. Standards and guidelines for the interpretation of sequence variants: a joint consensus recommendation of the American College of Medical Genetics and Genomics and the Association for Molecular Pathology. Genet Med. 2015 17(5):405-24.

© 2019 Cehajic-Kapetanovic J et al. *JAMA Network Open.*
